# Supplementary figures and images for: Association of tamoxifen resistance and lipid reprogramming in breast cancer
Source: BMC Cancer. 2018 Aug 24;18:850. doi: 10.1186/s12885-018-4757-z (PMC6109356; doi:10.1186/s12885-018-4757-z)

**A**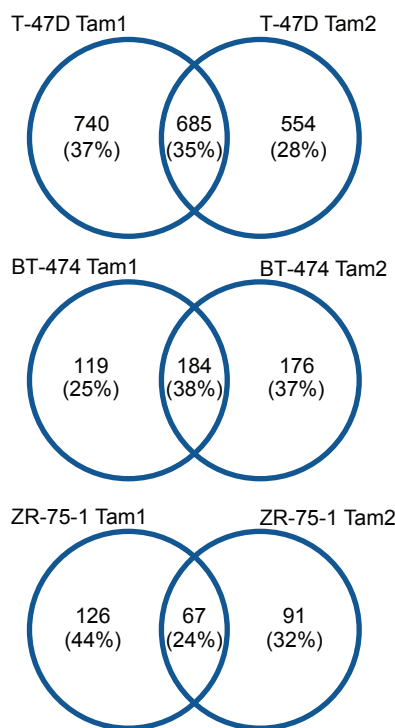**B**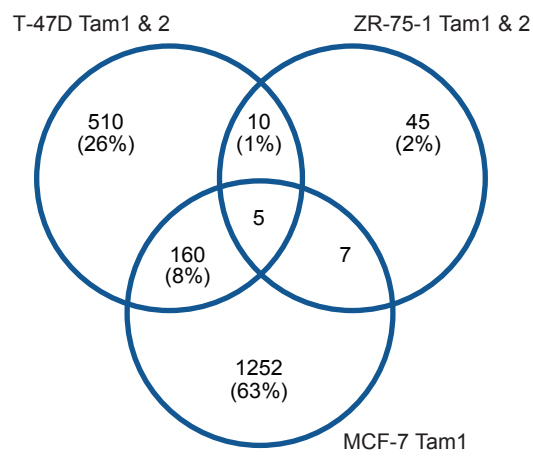

Supplement: Supplementary file 5 — Figure S1. Tamoxifen-resistant cell lines display distinct expression changes. Tamoxifen-resistant clones derived from same parental cells (A) and of the luminal A subtype (B) differ in their expression changes. Venn diagrams show overlap in numbers and percentage of genes that are differentially expressed. (PDF 363 kb) [file 12885_2018_4757_MOESM5_ESM.pdf]
